# Non-functioning parathyroid cyst presenting as a neck mass

**DOI:** 10.4322/acr.2024.505

**Published:** 2024-07-12

**Authors:** Sangamitra Rajasekaran, Adarsh Barwad, Suvradeep Mitra

**Affiliations:** 1 Post Graduate Institute of Medical Education and Research (PGIMER), Department of Histopathology, Chandigarh, India; 2 All India Institute of Medical Sciences (AIIMS), Department of Pathology, New Delhi, India

**Keywords:** Cysts, Parathyroid Glands, Parathyroid Hormone

Parathyroid cyst (PC) is an uncommon cause of neck mass and accounts for 0.8- 3.41% of parathyroid lesions. Females are more commonly affected than males (F:M- 2.5:1).^1^ Nearly 90% of PCs are non-functional, while the remaining are functional and secrete parathormone. Although functional PCs usually manifest cystic degeneration of a parathyroid adenoma, simple functional cysts of the parathyroid gland presenting features of hyperparathyroidism have also been documented.^2^ PC mainly develops in the inferior parathyroid glands, like the index case. The presentation may vary from asymptomatic neck mass to compressive symptoms such as hoarseness, dysphagia, or dyspnea. On examination, they are palpable as a soft, fluctuant cystic mass that moves with deglutition. Ultrasonogram usually reveals an anechoic thin-walled cyst with posterior enhancement, and computed tomography and magnetic resonance imaging demonstrate the cystic nature of the lesion and its anatomical relationship. Scintigraphy may not help determine the exact location of the cyst. Histopathological examination remains the gold standard for the diagnosis. The best treatment option for both functional and non-functional PC is surgical excision. Other options, such as simple aspiration and percutaneous injection of sclerosing agents, may also be attempted in cases of non-functional PC.^3^

Figure 1 refers to the case of a 24-year-old young female who presented with an asymptomatic neck swelling that progressively increased over the last 4 months. There were no complaints of dyspnea, dysphagia, palpitation, or symptoms of hypothyroidism. On clinical examination, a 5×4 cm swelling was palpable in the left anterior aspect of the neck. It was smooth with regular, well-defined margins, firm, and non-tender, moving with the deglutition but not with tongue protrusion. No lymphadenopathy was present. Lab investigations revealed normal thyroid hormone profile; triiodothyronine (T3: 0.82 ng/ml; RR: 0.35- 1.93 ng/ml), thyroxine (T4: 9 µg/dl; RR: 4.87-11.729 µg/dl), thyroid stimulating hormone (TSH:1.53 µIU/ml; RR:0.35-4.94 µIU/ml) and parathyroid hormone (PTH: 46.4 pg/ml; RR: 15-65 pg/ml). Contrast-enhancing computed tomography revealed a hypodense, well-defined cystic lesion of 72x46x40 mm on the left side of the thyroid with extra-thyroid extension inferiorly up to the manubrium sternum (Figure 1A). There was no internal calcification or solid component within the cyst. In addition, multiple sub-centimetric cervical lymph nodes in the upper, lower, mid-jugular region and posterior triangle of the neck were identified. Based on the clinical and imaging findings, a benign thyroid cyst was suspected in a euthyroid individual. The cyst’s fine needle aspiration cytology (FNAC) yielded 5 mL of clear fluid containing cholesterol crystals and occasional foamy macrophages in a fluidy background. A complete surgical excision was performed. Intraoperatively, a large cyst was identified arising from the lower pole of the left lobe of the thyroid, extending inferiorly up to the manubrium sternum, laterally up to the common carotid artery, and superiorly up to the hyoid bone. Grossly, the cyst was collapsed, was thin-walled, and had smooth outer and inner surfaces. No attached thyroid gland was identified. The wall showed uniform thickness (0.1-0.3cm). The capsular surface was smooth, and the cyst contained a brownish fluid. Microscopically, the cyst was lined by flat cuboidal to low columnar epithelium. The cyst wall showed discontinuous bands of parathyroid tissue embedded within the fibroconnective tissue (Figure 1B). These cellular foci were composed of lobules and organoid nests of monomorphic cells with central round nuclei, granular chromatin, inconspicuous nucleoli, and clear to pale eosinophilic cytoplasm (Figure 1C). No solid areas or features of adenoma were seen. On immunohistochemistry, these cells were positive for parafibromin (diffuse strong nuclear) (Figure 1D) and synaptophysin (diffuse strong cytoplasmic granular) and were negative for TTF-1, thyroglobulin, and calcitonin, confirming the diagnosis of PC. The right inferior parathyroid was normal.

**Figure 1:**
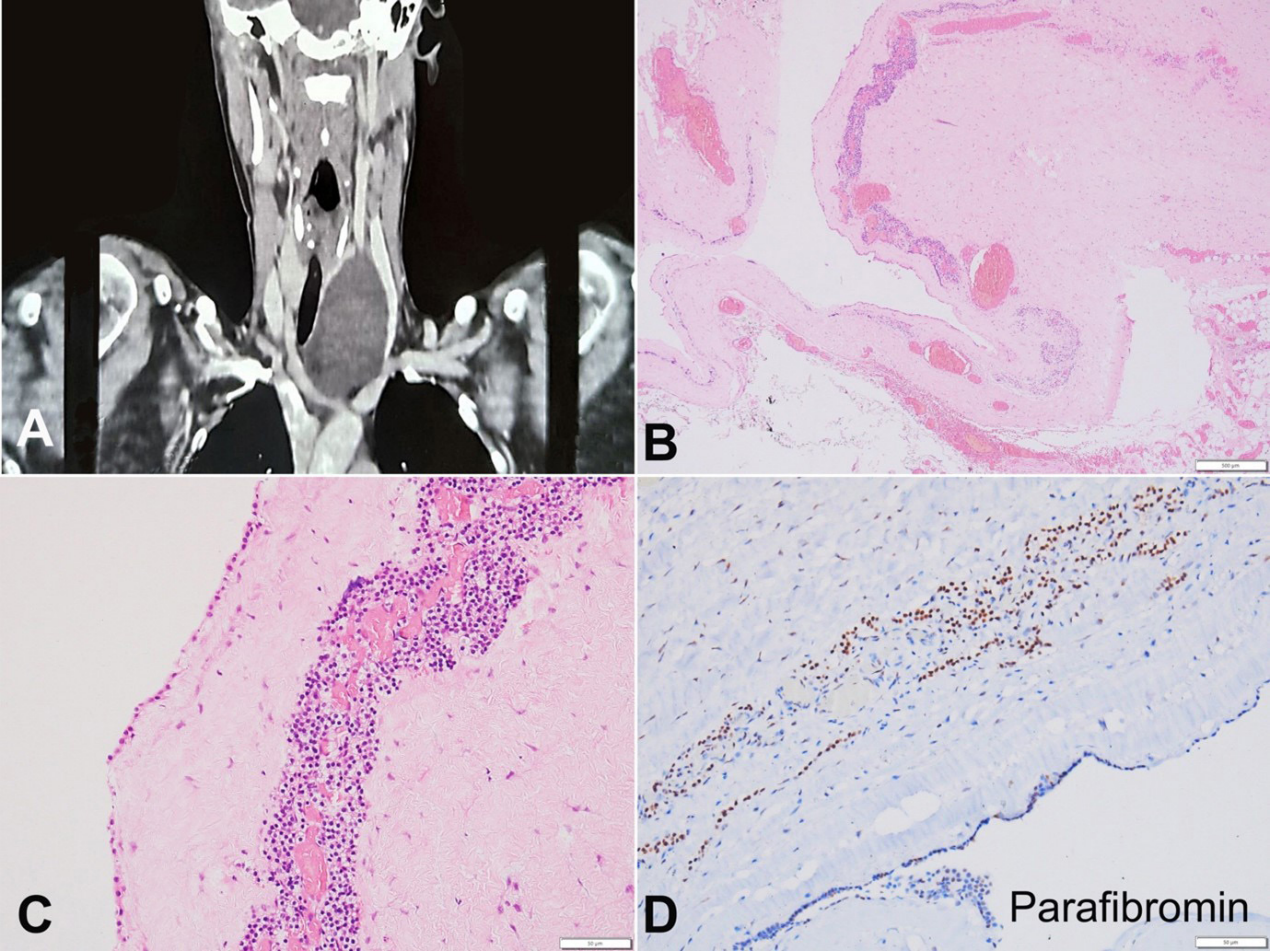
**A** – CECT image showing hypodense cystic lesion in the left side of neck with extra thyroid extension inferiorly up to the manubrium sternum; **B-D** – Photomicrographs of the cystic lesion; **B** – Cyst showing lobules of cells embedded within the collagenous wall underneath the lining epithelium (H&E; 40X); **C** – Flat cuboidal lining epithelium and aggregates of monomorphic parathyroid cells with optically clear cytoplasm in the cyst wall (H&E; 400X); **D** – Strong nuclear positivity for Parafibromin immunostain (200X).

## References

[1] Uehara A, Suzuki T, Yamamoto Y (2020). A functional parathyroid cyst from the hemorrhagic degeneration of a parathyroid adenoma. Intern Med.

[2] Silva R, Cavadas D, Vicente C, Coutinho J (2020). Parathyroid cyst: differential diagnosis. BMJ Case Rep.

[3] Xu P, Xia X, Li M, Guo M, Yang Z (2018). Parathyroid cysts: experience of a rare phenomenon at a single institution. BMC Surg.

